# Clinical Application Study of Minimally Invasive Double-Reverse Traction in Complex Tibial Plateau Fractures

**DOI:** 10.1155/2022/5564604

**Published:** 2022-01-22

**Authors:** Faqi Cao, Hang Xue, Chenchen Yan, Ze Lin, Bobin Mi, Adriana C. Panayi, Tian Xia, Wu Zhou, Hui Li, Guohui Liu

**Affiliations:** ^1^Department of Orthopedics, Union Hospital, Tongji Medical College, Huazhong University of Science and Technology, 1277 Jiefang Avenue, Wuhan, 430022 Hubei, China; ^2^The Division of Plastic Surgery, Brigham and Women's Hospital, Harvard Medical School, Boston, MA, USA

## Abstract

The aim of this study was to evaluate the clinical application of double-reverse traction for minimally invasive reduction of complex tibial plateau fractures. A retrospective analysis was performed to identify all patients admitted to the Department of Orthopedics, Union Hospital, Tongji Medical College, Huazhong University of Science and Technology, from March 2017 to December 2019 with Schatzker type VI tibial plateau fractures. 12 patients were identified (7 men and 5 women) with an average age of 46.15 ± 13 (39-58) years old. All patients were treated with double-reverse traction and closed reduction. After the fracture was reduced, the bone plate was fixed by percutaneous minimally invasive implantation. Outcomes assessed in this study include operation time and intraoperative blood loss. Imaging was performed during the postoperative follow-up, and functional recovery was evaluated at the final follow-up according to the Hospital for Special Surgery (HSS) score and the International Knee Joint Literature Committee (IKDC) functional score. Patients were followed up for 12.54 ± 1.5 (8-15) months. The average operation time was 63.63 ± 21 (35-120) minutes, and the average intraoperative blood loss was 105.45 ± 21 (60-200) mL. The Rasmussen imaging score was either excellent or good in all cases. The knee joint HSS score was 86.15 ± 6 (79-90) points, and the IKDC score was 80.01 ± 11 (75-90) points. No complications, such as wound infection, incision disunion, loosening of internal fixation, and internal fixation failure, occurred. In the treatment of Schatzker VI type complex tibial plateau fracture, the dual-reverse traction minimally invasive technique has the advantages of safety and effectiveness, less soft tissue injury, and allowing early joint movement, which is worthy of clinical promotion.

## 1. Introduction

Tibial plateau fractures are common intra-articular fractures in the adult population, accounting for 1.66% of total body fractures. The Schatzker classification is currently one of the most widely implemented clinical fracture classifications [1]. Schatzker II-VI fractures account for 85.85% of tibial plateau fractures, with Schatzker type VI fractures, which are caused by high-energy injuries, often causing severe peripheral soft tissue damage, joint collapse, and tibial condylar separation. Successful treatment of such fractures is a notable challenge in the field of orthopedic trauma [2]. The current conventional treatment of tibial plateau fractures consists of conservative management, internal fixation, external fixation, and joint replacement. Moreover, as for Schatzker type VI tibial plateau fractures, the current mainstream approach mainly involves double-incision and double-plate therapy accompanied by arthroscopic treatment. However, when patients receive such conventional treatments, they may suffer iatrogenic trauma, which aggravates soft tissue damage and increases the incidence of complications such as postoperative infection [3]. The prognostic complications mentioned above all cause great difficulties for clinicians and need to be fixed urgently. Therefore, minimally invasive reduction and fixation offers great potential for the treatment of such fractures. The double-reverse traction repositor (DRTR), developed by Professor Yingze Zhang, has shown significant advantages in the treatment of complex tibial plateau fractures. This surgical method, noted to be simple and quick, allows for minimally invasive reduction and fixation with few postoperative complications. In the current study, we assess the outcomes of 12 patients with complex type VI tibial plateau fractures who were treated with the DRTR. The purpose of this study is to assess the efficacy of the DRTR surgical technique as a minimally invasive treatment of type VI tibial plateau fractures and to demonstrate the safety and efficacy of this technique by roughly comparing it with those associated with common-sense open internal fixation [4].

## 2. Materials and Methods

### 2.1. Patient Information

We retrospectively analyzed 12 patients (7 men and 5 women) with Schatzker type VI tibial plateau fractures who had been admitted to the Department of Orthopedics, Union Hospital, Tongji Medical College, Huazhong University of Science and Technology, from March 2017 to December 2019. The average age of the patients was 46.15 ± 13 (39-58) years old. Cause of injury included motor vehicle accidents (6 patients), electric vehicle falls (4 patients), and crush injuries (2 patients). All patients underwent relevant assessment during admission to exclude surgical contraindications. The average time from injury to operation was 8.52 ± 3 days. The classification of the damage of the soft tissue adopted AO-IC, IC-1(3 patients), IC-2(8 patients), and IC-3(1 patient), (Table 1).

### 2.2. Inclusion and Exclusion Criteria

We included all patients: (1) between 18-65 years, (2) with Schatzker VI tibial plateau fractures, (3) with injuries due to high-energy velocity and torsion, (4) with normal lower limb function prior to injury, (5) who had a closure time from injury to surgery of less than 3 weeks patients, (6) with no other peri-knee fractures, and (7) who agreed to receive minimally invasive treatment with double-reverse traction.

Patients excluded were those (1) with open, knee degeneration, pathological fractures, or severe knee joint degeneration; (2) with a history of peripheral nerve, vascular injury, and compartment syndrome; and (3) with poor general condition or with severe systemic diseases.

This study has been approved by the Medical Ethics Committee of the Union Hospital, and informed consent was obtained from all included patients.

### 2.3. Method of Operation

**Figure 1:**
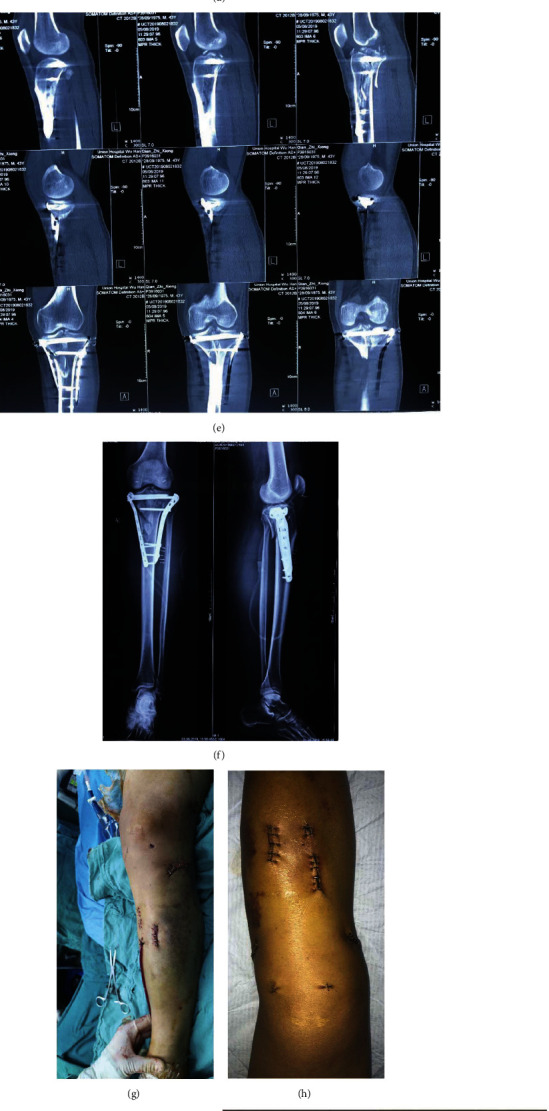
A 43-year-old male with Schatzker type VI tibial plateau fracture with spur sign. (a, b) X-rays of tibial plateau fracture presurgery. (c–e) Preoperative CT imaging results of the tibial plateau fracture. (f) X-rays of tibial plateau obtained after operation. (g, h) Intraoperative and 3 days postoperative incisions of the entire injured limb.(i) Application of double-reverse traction repositor (DRTR) during operation. (j–m) Intraoperative fluoroscopic X-ray images of the fracture sites before and after the application of the traction device.

**Figure 2:**
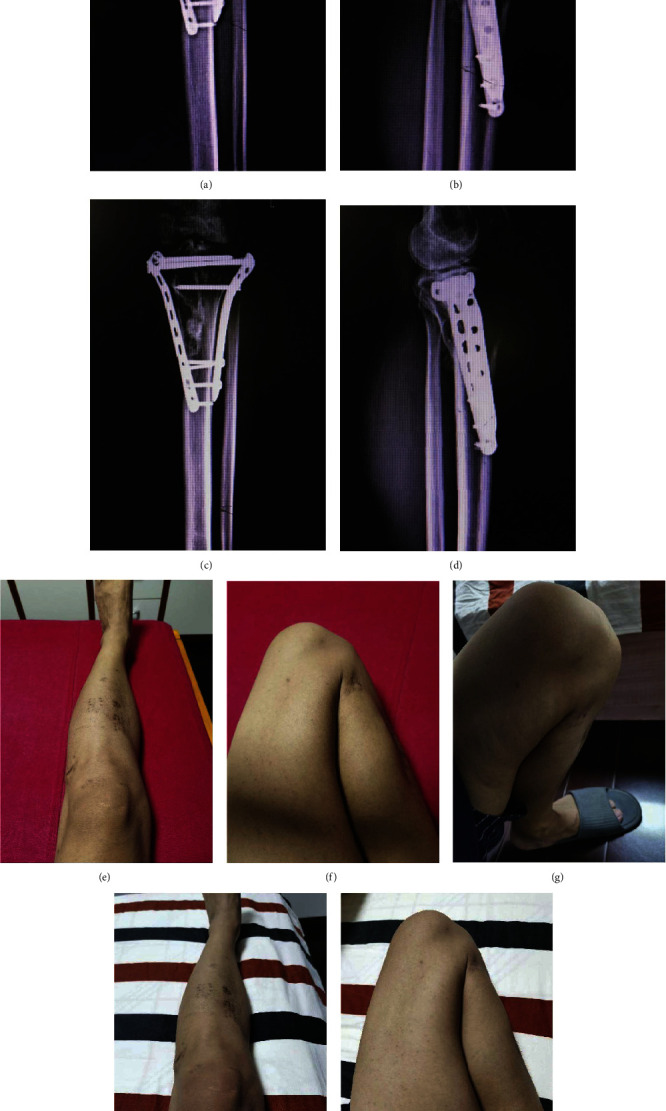
Follow-up results of the 43-year-old male mentioned in Figure 1 were obtained at 6 and 13 months after operation. (a, b) X-rays of tibial plateau obtained 6 months after operation. (c, d) X-rays of tibial plateau obtained 13 months after operation. (e, f) Photographs of the knee joint in extended and flexed positions 6 months after operation. (g–i) Photographs of the knee joint in extended, flexed, and feather-weightbearing positions 12 months after operation.

**Figure 3:**
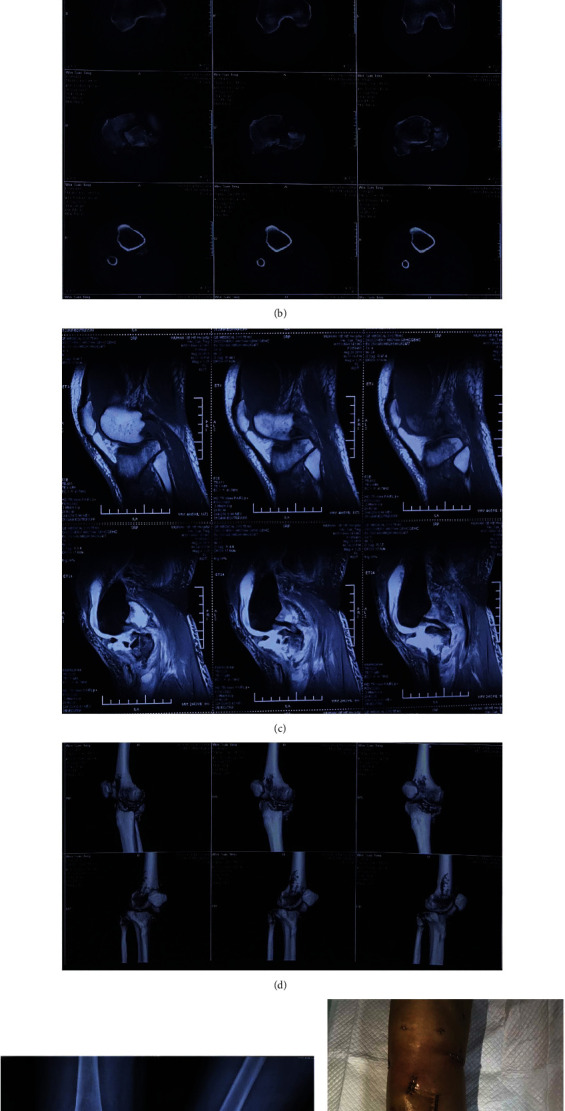
A 55-year-old male as a typical case of articular surface collapse. (a) X-rays of tibial plateau obtained before operation. (b) CT of tibial plateau obtained before operation. (c) MRI of the knee joint before operation. (d) CT of tibial plateau in 3D before operation. (e) X-rays of tibial plateau obtained right after operation. (f) Postoperative appearance of the affected limb.

All surgeries were performed by the chief physician. Surgery was initiated after satisfactory general anesthesia. The patient was placed in the supine position. *φ*2.5 mm Kirschner wires were placed on the femoral condyle and the distal tibia. Two traction bows were placed on the Kirschner wire and connected with the double reverse traction repositor. Bone traction must be performed along the long axis of the tibia and extended until the calf muscles are tightened. Reduction of the fracture block was observed under C-arm digital subtracting X-ray system. The force line of the knee joint was adjusted with the double-reverse traction device restoring the length and force line of the front and lateral tibia under C-arm digital subtracting X-ray system. The gap on the outer platform was retracted. The articular surface of the distal femur was used as a guide to restore the articular surface of the tibial plateau during the reduction process. If the articular surface was collapsed, a 2.5 mm guide pin was inserted under fluoroscopy from the inner and lower part of the tibial tubercle about 5 cm below and 1 cm behind the tibial spine. If the medial fracture line involved the positioning point, it could be lowered appropriately. The guide needle was pushed forward to 1 to 2 cm below the collapsed bone mass and, based on the guide pin, a ring drill or a hollow gradient drill to gradually open to about 1.5 cm. Using the self-developed collapsed bone block top rod system to insert into the bone tunnel, adjust the angle and gently tap the end of the top rod under fluoroscopy to gradually lift the collapsed fracture block up and restore the flatness of the articular surface. A bicortical iliac strip of appropriate size is removed from the autogenous iliac bone to support the bone graft through the bone tunnel. After bone grafting, a minimally invasive incision is created and the lateral plate is inserted. A drill is placed horizontally at the proximal end for temporary fixation, a locking screw is placed at the distal end, and the medial plate is inserted. The squeezed bolts are inserted into the positioning holes on the outer plate depth, which is required to determine the length prior to insertion, and the width of the joint surface is reset. Performer adjusts the position of the inner plate on the opposite side to ensure that the bolts pass through the inner plate nail holes. The bolts are tightened, and the tail broken. Finally, screw in the remaining screws of the medial and lateral plates in turn removes the traction frame, grinds the articular surface over the flexion position, and strikes the heel in the extension position to further flatten the articular surface. Routine postoperative arthroscopic exploration showed that the articular surface fractures were reduced well with the step smaller than 2 mm (probing hook diameter). Typical case is shown in Figures 1, 2, and 3.

Patients received routine anticoagulation and detumescence treatment 24 hours after surgery. The quadriceps muscle function was passively exercised on the first day after surgery. The drainage tube was removed on the second day after surgery. After the sixth week, patients were able to start ambulating without weight-bearing. 12 weeks after surgery, depending on the healing condition of the fracture, patients were gradually able to walk with weight.

### 2.4. Follow-Up and Observation Indicators

The patients were followed up for 1, 3, and 6 months after surgery, and imaging was performed. Final follow-up imaging was scored according to the Rasmussen evaluation standard. The functional recovery of the affected knee joint was evaluated using the New York Special Surgery Hospital (HSS) score and the International Knee Documentation Committee (IKDC) functional score. Postoperative complications were monitored, and intraoperative data such as operation time and intraoperative blood loss were collected.

### 2.5. Statistical Methods

Data entry and statistical analysis were performed using SPSS13.0 (SPSS Corporation, USA) statistical software. The Kolmogorov-Smirnov test was used to verify whether distribution conforms to normal distribution. The age of patients, time from injury to operation, follow-up time, operation time, and HSS and IKDC scores are expressed as *xˉ* ± *s*. The *α* value of the inspection level was set to 0.05.

## 3. Results and Discussion

All patients underwent successful surgical treatment, and there was no attrition to surgical follow-up. The follow-up time was 8-15 months, with an average of 12 months. One patient had obvious skin contusion before operation and epidermal necrosis after operation which improved with dressing change. All other patients showed no complications such as wound infection and nonunion after operation. Postoperative X-ray imaging in the double-reverse traction group showed satisfactory fracture reduction and proper position of the bone plate and screws. The average operation time was 63.63 ± 21 (35-120) min, and the average intraoperative blood loss was 105.45 ± 21 (60-200) mL. At the final follow-up, all patients scored excellent or good on the Rasmussen imaging score. The average knee HSS score was 86.15 ± 6 (79-90) points, and the average IKDC score was 80.01 ± 11 (75-90) points. Lower limb length difference was 1.12 ± 0.58 (0-2.21) cm.

Tibial plateau fractures are intra-articular fractures with high incidence. Schatzker VI fracture is the most severe type of tibial plateau fracture. It is mostly caused by high-energy impact injuries such as vehicle accidents and falls from heights. Schatzker type VI fractures usually manifest as comminuted fractures which involve the entire tibial condyle and articular surface [5]. They are often accompanied by damage to the cruciate ligament and meniscus, causing serious changes in the normal anatomical relationship of the tibial condyle [6]. Following a Schatzker type VI fracture, the soft tissue around the knee joint has relatively poor blood supply, which can result in wound infection and skin necrosis and even hinder bone union. Current treatment for Schatzker type VI fractures is limited to incision reduction, rigid internal fixation, and early functional exercise. However, the traditional open reduction and internal fixation surgery requires long incisions, large soft tissue damage, long operation time, and large dissection range, and, hence, the incidence of postoperative complications is also higher [7]. The literature supports that maintaining the overall force line of the lower limbs is more important than anatomical reduction of the articular surface. Recovery of the knee joint stability is an important factor affecting the long-term efficacy of tibial plateau fracture treatment [8]. Rehabilitation does not entirely depend on the anatomy of the articular surface. Satisfactory results can be obtained with repositioning if the overall force line of the lower limbs recovers well, even if part of the articular surface is uneven. Koval et al. showed that there was no significant correlation between the reduction of the articular surface as shown through imaging and clinical efficacy.

With the development of biomechanics and their clinical application, it has become evident that fracture treatment should not only aim to restore anatomical position but also protect the soft tissue. Treatment of fractures does not only restore the anatomical position but also allows protection of the soft tissues [9]. This is particularly important in fractures like Schatzker VI tibial plateau, in which the soft tissue around the knee joint is seriously injured, the skin tension is high, and tension blisters easily form, and clinical treatment is more challenging. If the traditional internal and external steel plate is used for fixation, the surgical incision is long, and trauma to the tissue is extensive [10]. A clinical case pointed out that DRTR has achieved good results in the treatment of complex tibial plateau fractures [11]. In addition, studies have also pointed out that DRTR has the advantages of soft tissue damage when dealing with high-energy impact injuries of the knee joint [12]. Concurrently, blood supply to the anterior tibia is poor, and complications such as skin necrosis can easily occur after surgery. The use of minimally invasive technology in the field of orthopedics is receiving more and more attention. The goal of this technology is to minimize soft tissue damage, preserve the blood supply of the fracture as much as possible, and reduce iatrogenic injury. With extensive experience in clinical practice, Professor Yingze Zhang creatively put forward the theory of “homeopathic reduction” of fractures, whereby he recommends conforming to the mechanical axis of the limbs and the soft tissue movement trajectory and resetting the fracture ends through traction. In the surgical treatment of tibial plateau fractures, restoring the lower limb force line and height of the collapsed articular surface while maintaining the stability of the plateau fracture after reduction is the focus of treatment. The double-reverse traction device developed by Professor Yingze Zhang uses the self-traction of the ligaments around the knee joint and the joint capsule to achieve the reduction and maintenance effect. At the same time, it provides effective traction and can maintain the reduction of the fractured end. This is particularly useful in fractures that require continuous traction and reduction [13].

In this study, the different positions of the double-reverse traction distal device were adjusted to restore the force line of patient's lower limbs, correct the knee joint varus, and effectively maintain the fractured end [14]. Furthermore, the joint was not opened during the operation, avoiding iatrogenic injury of the knee ligament and joint capsule, which is beneficial for the stability and functional recovery of the knee joint. In patients with obvious articular surface collapse that cannot be recovered, the medial tibial bone tunnel is established ,and the self-made collapsed bone block top rod system is inserted into the bone tunnel. The end of the top rod is gently tapped under fluoroscopy to adjust the angle in order to achieve a gradual lift of the collapsed fracture block, thereby restoring the flatness of the articular surface. An appropriate bicortical iliac strip must be removed from the autogenous iliac bone to support the bone graft in the bone tunnel. In line with the principles of tibial plateau fracture surgery, this procedure firmly fixes the fracture and allows early nonweight-bearing knee exercises and improved limb movements [15]. The minimally invasive treatment concept enabled by the “Zhang Traction Frame” should be a goal pursued both by doctors and patients as, in comparison to the commonly used traction beds in clinical practice, it has great advantages. In addition, it is helpful for large-scale promotion and application in hospitals at all levels across the country. A disadvantage of our study is the fact that it is retrospective, the number of cases is small, and the patients included in the study had no serious other compound injuries that may have caused delays in surgical treatment. In addition, the follow-up time is not very long, and further follow-up of the long-term treatment effect of the operation is needed to evaluate the incidence of osteoarthritis and other data. In a future cohort study, we aim to observe and follow up more cases [16].

In summary, for the treatment of Schatzker type VI tibial plateau fractures caused by high-energy impact injuries, double-reverse traction and minimally invasive closed reduction and internal fixation can achieve satisfactory results in a short period of time. Compared with traditional surgical methods, this technique has obvious advantages and is worthy of increased clinical application.

## 4. Conclusions

In patients with Schatzker VI complex tibial plateau fractures, minimally invasive double-reverse traction is a treatment method with short operation time, minimal bleeding, quick postoperative recovery, low infection rate, and satisfactory clinical efficacy.

## Figures and Tables

**Table 1:** Basic information of included patient.

Group	Number of cases	Gender	Cause of injury	Age	Time before operation	Classification of soft tissue
	Cases	Male/female	Vehicle accidents/fall injury/crush injuries	Years	Days	IC1/IC2/IC3
DRTR	12	7/5	6/4/2	46.15 ± 13	8.52 ± 3	3/8/1

The data included is of patients who were available at 12 months.

## Data Availability

The data used to support the findings of this study are available from the corresponding author upon request.
